# Development and content validation of a risk classification instrument

**DOI:** 10.1590/0034-7167-2023-0502

**Published:** 2024-09-06

**Authors:** Marcia Beatriz Micha Ferreira de Oliveira, Lillian Caroline Fernandes, Ilana Eshriqui Oliveira, Ramon Antônio Oliveira, Flávio Rebustini, Ana Carolina Cintra Nunes Mafra, Eduarda Ribeiro dos Santos

**Affiliations:** I Sociedade Beneficente Israelita Brazileira Albert Einstein, Faculdade Israelita de Ciências da Saúde Albert Einstein. São Paulo, São Paulo, Brazil; II Universidade de São Paulo, Escola de Enfermagem, Departamento de Enfermagem Médico-Cirúrgica. São Paulo, São Paulo, Brazil; III Sociedade Beneficente Israelita Brazileira Albert Einstein, Centro de Estudos, Pesquisa e Práticas em Atenção Primária à Saúde e Redes (CEPPAR). São Paulo, São Paulo, Brazil; IV Universidade de São Paulo, Escola de Ciências, Humanidades e Artes, Departamento de Gerontologia. São Paulo, São Paulo, Brazil; V Hospital Sírio Libanês. São Paulo, São Paulo, Brazil

**Keywords:** Triage, Validation Studies, Psychometrics, Patient Acuity, Primary Health Care, Medición de Riesgo, Estudio de Validación, Psicometría, Gravedad del Paciente, Atención Primaria de Salud

## Abstract

**Objective:**

Develop and validate the content of an instrument for patient risk classification in emergency services of Primary Health Care.

**Method:**

The study included two stages: item generation and content validity. A literature review and retrospective analysis of medical records were conducted to create the instrument items. The Content Validity Ratio (CVR) was used to assess agreement among judges during content validation.

**Results:**

In the first and second rounds, 75 and 71 judges validated the risk classification instrument, respectively. The minimum adherence score for the latent variable item based on the final number of judges was 0.22 and 0.18; thus, 52 items, divided into three classification categories (red, orange, and yellow), were retained.

**Conclusion:**

The instrument was considered valid regarding clarity, relevance, pertinence, and agreement regarding the severity indicated in the item.

## RESUMEN

**Objetivo:**

Desarrollar y validar contenido de un instrumento para medición de riesgo del paciente en servicios de emergencia de Atención Primaria de Salud.

**Método:**

Estudio compuesto por dos etapas: generación de ítems y validez de contenido. Realizada revision de literatura y análisis retrospectivo de prontuarios para creación de los ítems del instrumento. En la validez de contenido, fue usado el Content Validity Ratio (CVR) para averiguar la concordancia entre jueces.

**Resultados:**

El instrumento de medición de riesgo fue validado por 75 y 71 jueces en el primer y segundo ciclo, respectivamente. Utilizada calificación mínima de adherencia al ítem de la variable latente basado en el número final de jueces, adoptando 0,22 y 0,18; y mantenidos 52 ítems divididos en tres categorías de medición (rojo, naranja y amarillo).

**Conclusion:**

El instrumento fue considerado válido cuanto a claridad, relevancia, pertinencia y concordancia en relación con la gravedad indicada en el ítem.

## INTRODUCTION

Patient reception is defined as a health practice that involves qualified listening and aligning user demands with the service’s response capabilities, a task to be performed by all healthcare professionals. In contrast, Risk Classification (RC) is a technical and care-related work process that ensures timely care following a pre-established protocol. In Brazil, Resolution 423/2012 of the Federal Nursing Council states that RC is an exclusive activity of professional nurses^(1, 2, 3)^.

The high volume of care and subsequent overcrowding of urgency and emergency services are issues discussed both nationally and internationally^(4, 5)^. Thus, there are ongoing discussions about the Health Care Network (RAS), particularly concerning Primary Health Care (PHC), which serves as both the entry point and the element that organizes and coordinates the Urgency and Emergency Care Network. In this context, RC protocols are recognized as part of assertive resolutions related to initial care, making their implementation in RAS emergency services essential^(5, 6)^.

Globally, there is a recommendation to use protocols that stratify the clinical severity of patients. The most commonly used include the Australasian Triage Scale (ATS), Canadian Triage and Acuity Scale (CTAS), Emergency Severity Index (ESI), and predominantly the Manchester Triage System (MTS)^(7, 8, 9, 10, 11, 12, 13)^. The MTS has become the most applied in urgency and emergency services at all levels of care; however, over time, some cost-related barriers have been identified in Brazil, as its implementation and maintenance require specific training with the Brazilian Group of Risk Classification^(13)^.

Studies have shown that the MTS effectively predicts outcomes in hospital emergency services^(14, 15)^. However, research conducted in primary care institutions revealed weaknesses^(16, 17)^. An experience report highlighted that because of the MTS being developed in a hospital setting, factors such as low clinical complexity and material resources in primary care institutions hinder its applicability, potentially necessitating adaptations^(18)^. This finding was supported by a scoping review^(19)^.

Given this context, considering that instruments currently used for risk classification are developed and validated specifically for each country’s scenario and focused on hospital care, it is necessary to develop and validate a risk classification instrument for use in PHC emergency services within the Unified Health System (SUS)^(13, 14, 15)^.

## OBJECTIVE

To develop and validate a risk classification instrument for use in PHC emergency services.

## METHODS

### Ethical Aspects

This study was approved by the Research Ethics Committee involving Human Beings of the Municipal Health Department of São Paulo and the Research Ethics Committee involving Human Beings of the Sociedade Beneficente Israelita Brasileira Albert Einstein via the Brazil platform. All participants signed the Informed Consent Form.

### Study Design

This is a psychometric study consisting of two stages: item generation and content validity through a panel of experts. The stages included defining objectives, constructing items, selecting and organizing items, structuring the instrument, obtaining expert opinions, and validating the content. This research was structured according to the Revised Standards for Quality Improvement Reporting Excellence (SQUIRE 2.0)^(20)^.

### Stage 1: Literature Review and Item Development

For constructing the instrument, a narrative review was conducted in PubMed, LILACS, Scopus, and SciELO databases using terms combined with the Boolean operator “OR”: Triage, Risk Classification, HumanizaSUS, and Manchester. Additionally, an exploratory retrospective analysis of the demands of PHC emergency services was performed. The PHC in São Paulo consists of 450 Primary Health Units (UBS) and 117 Ambulatory Medical Assistance units (AMA), of which 87 are integrated with UBS^(21)^.

In São Paulo, emergency consultations accounted for 43% of all SUS user consultations in 2005; however, most of these attendances were of complexity compatible with UBS and did not require emergency room and hospital infrastructure. In response, in 2009, the Municipal Health Department proposed the creation of AMAs linked to primary care, aimed at increasing the population’s access to the basic health network^(22)^.

Considering these definitions, the demands of three AMA units totaling 358,555 attendances in 2022 were analyzed. The main complaints, signs, and symptoms related to each severity classification, along with the outcomes of death and transfer, were examined. Categorical variables were described by absolute frequency and percentages, while numerical variables were described by mean and standard deviation or medians and quartiles, depending on the data distribution. The relationships between each variable obtained in RC and outcomes were analyzed using hypothesis tests and correlation coefficients, depending on the data nature. Analyses were conducted utilizing R Core Team software with a 5% significance level.

At the end of this stage, it was found that most emergency attendances in PHC were in the green and blue categories, where, according to current protocols, patients could wait 120 and 240 minutes, respectively. To ensure shorter waiting times, the present risk classification instrument divided signs and symptoms into three categories: red (immediate care), orange (up to 10 minutes care), and yellow (up to 60 minutes care). Risk classification instruments typically have five severity categories: red, orange, yellow, green, and blue, suggesting waiting times of 0, 10, 50, 120, and 240 minutes, respectively.

### Stage 2: Content Validation

Inclusion criteria were: i) being a doctor or nurse with at least five years of experience, and ii) working in Brazilian public urgency and emergency services; intensive care units; PHC; or university teaching with research experience in urgency and emergency care. Experts were identified and selected using the Curriculum Lattes system, applying filters for the professional practice area and selecting the major area – health sciences; the area – nursing and medicine; and the subarea – “urgency and emergency” and “emergency.”

The invitation for both rounds was conducted using the “snowball” technique^(23)^, contacting eligible professionals from all Brazilian states who could indicate other potential judges. The link with the invitation to participate as a judge was sent via social media groups and the websites of the Federal and Regional Nursing Councils – São Paulo. The validation process occurred in two periods: the first round from January to March 2021 and the second round from July to September 2021.

### Data Collection

After verifying eligibility criteria, the TCLE was sent via email along with a link to the CR instrument evaluation form, accompanied by detailed information and/or images on the proposed items for validation (supplementary material). Data collection was conducted through RedCap®.

Judges evaluated the items based on: i) clarity, ii) relevance, iii) pertinence, and iv) agreement regarding the severity classification of the item. They could also suggest wording changes for each item.

Before the questionnaire, affirmatives were provided for each item: i) “The item is clear: it is possible to identify what needs to be evaluated based on how it is written”; ii) “The item is relevant for measuring patient severity: it is crucial for the risk classification assessment”; iii) “The item is measurable by the nurse: in practice, the nurse can assess this item during risk classification”; iv) “This item needs to change classification: the item is relevant and pertinent but is in the wrong classification. If you want to leave a comment or suggest a change, click ‘comment’ to open a text field for each item at the end of each classification.” A “yes” or “no” layout was used for evaluating all items in each domain. The questionnaire was considered valid when all items and domains had responses.

### Data Analysis

The Content Validity Ratio (CVR) method was used, proposing to validate the items based on agreement from a wide panel of experts on each evaluated criterion, ranging from -1 (perfect disagreement) to 1 (perfect agreement)^(18, 19)^. Concordance coefficients were compared to the classification in Altman, considering coefficients below 0.2 as poor, between 0.2 and 0.4 as fair, between 0.4 and 0.6 as moderate, between 0.6 and 0.8 as good, and above 0.8 as excellent. The minimum adherence score of the item to the latent variable was based on the critical CVR values, with 0.22 as the cutoff point in the first round and 0.18 in the second to consider the item satisfactory in terms of content validity^(18, 19)^. Statistical analyses were conducted using the Statistical Package for the Social Sciences (SPSS).

## RESULTS

## Stage 1: Characterization of Judges

The proposed instrument was evaluated in two rounds until achieving the necessary consensus level. Table 1 describes the profile of participating judges.

**Table 1:** Characterization of Expert Judges Participating in the Two Rounds, São Paulo, Brazil, 2021

Characterization Data	First Round (n = 75)	Second Round (n = 71)
Mean age in years (SD)	39.95 (8.00)	42 (8.00)
Categorical variables: no (%)		
Sex		
Female	54 (72.00)	48 (67.60)
Male	21 (28.00)	23 (32.40)
Profession		
Physician	15 (20.00)	11 (15.50)
Nurse	60 (80.00)	60 (84.50)
Highest educational level		
Undergraduate degree	8 (10.67)	9 (12.70)
Residency	4 (5.33)	2 (2.80)
Specialization	49 (65.33)	43 (60.60)
Master’s degree	6 (8.00)	12 (16.90)
Doctorate	6 (8.00)	5 (7.00)
Post-doctorate	2 (2.67%)	0 (0.00)
Brazilian region of professional activity		
North	1 (1.33)	1 (1.40)
Northeast	1 (1.33)	2 (2.80)
Midwest	6 (8.00)	2 (2.80)
South	11(14.67)	10 (14.10)
Southeast	56 (74.67)	56 (78.90)
Area of practice or research*		
Urgency and Emergency	38 (50.67)	42 (59.20)
Primary Care	40 (53.33)	29 (40.80)
Nursing Care in a hospital setting	16 (21.33)	24 (33.80)
Obstetrics	1 (1.33)	2 (2.8)
Pediatrics	12 (16.00)	5 (7.0)

*It was possible to select more than one option; SD – standard deviation.

## Stage 2: Construction of items

After the literature review and analysis of data from the exploratory retrospective stage, considering the most frequent complaints and those related to outcomes of transfer, death, and discharge, the instrument was composed of 94 items distributed across three severity categories (red, orange, and yellow). In the first round, all items had a CVR above the critical value for the Clarity domain, but other items and domains did not reach the cutoff point (Table 2).

**Table 2:** First Round of Evaluation of Items in the Urgency and Emergency Risk Classification Instrument (n = 75). São Paulo, Brazil, 2021

Risk Classification Instrument Items	Clarity (CVR)	Relevance (CVR)	Pertinence (CVR)	Concordance with Classification (%)
Red Category				
More than 25% of body surface area burned	0.41	−0.07	−0.17	98.70
Traumatic amputation	0.68	−0.17	−0.23	98.70
Baby being born	0.73	−0.33	−0.23	98.70
Spinal column compromise	0.41	−0.12	−0.52	97.30
Convulsing	0.87	−0.23	−0.25	100.00
Unresponsive child	0.79	−0.17	−0.33	98.70
Open fracture of large parts	0.68	−0.15	−0.28	100.00
Witnessed gastrointestinal hemorrhage	0.55	−0.17	−0.28	100.00
Otorrhagia (Battle’s sign – Raccoon eyes)	0.49	−0.12	−0.31	100.00
Facial burns involving airways	0.73	−0.09	−0.23	100.00
Second- and third-degree burns in the perineal area	0.63	−0.20	−0.23	100.00
Local burns with exposure of systems or organs	0.65	−0.17	−0.28	100.00
Ineffective breathing	0.55	−0.17	−0.28	96.00
Uncontrollable bleeding	0.65	−0.15	−0.28	98.70
Severe trauma	0.49	−0.12	−0.25	100.00
Non-patent airway	0.71	−0.15	−0.31	100.00
Orange Category				
Bulging fontanelle	0.68	−0.07	−0.20	96.00
Psychomotor agitation	0.60	−0.09	−0.12	98.70
Sudden visual alteration	0.57	−0.07	−0.33	96.00
Neurological changes within 24 hours	0.55	−0.15	−0.28	96.00
Amputation of small parts	0.60	−0.04	−0.12	94.70
Avulsion of permanent tooth within two hours	0.36	−0.23	−0.31	93.30
Bladder distension	0.55	−0.20	−0.12	93.30
Prostrate child	0.52	−0.12	−0.15	97.30
Evident respiratory distress	0.60	−0.01	−0.20	92.00
Decreased fetal movements	0.60	−0.17	−0.12	94.70
Precordial pain	0.63	−0.04	−0.31	89.30
Enterorrhagia	0.63	−0.09	−0.25	98.70
Between 24% and 15% of body surface area burned	0.55	−0.12	−0.20	96.00
Witnessed epistaxis	0.65	−0.12	−0.07	98.70
Postictal state	0.41	−0.07	−0.28	96.00
Vaginal bleeding in pregnant women over 20 weeks	0.60	−0.20	−0.20	96.00
Intense vaginal bleeding	0.39	−0.25	−0.12	88.00
Small part fracture	0.49	−0.09	−0.36	97.30
Ketotic breath	0.71	−0.09	−0.20	97.30
Inhalation of chemicals	0.63	−0.04	−0.36	97.30
Smoke inhalation	0.68	0.01	−0.33	96.00
Insertion of potentially dangerous object	0.52	−0.17	−0.23	93.30
Pelvic instability	0.49	−0.04	−0.39	89.30
Alcohol intoxication	0.55	−0.09	−0.23	97.30
Drug intoxication	0.57	−0.09	−0.28	97.30
Chemical intoxication	0.60	−0.12	−0.31	96.00
Lacerating injury	0.47	−0.01	−0.25	93.30
Chemical injury	0.55	−0.01	−0.25	96.00
Known lethal venom injury	0.60	−0.01	−0.41	88.00
Arterial or venous vascular injury	0.55	−0.12	−0.39	94.70
Melena	0.60	−0.15	−0.33	98.70
Meningism	0.60	−0.04	−0.15	96.00
Severe ear pain	0.63	−0.01	−0.20	96.00
Amniotic fluid loss	0.55	−0.25	−0.20	94.70
Associated precordial pain	0.41	−0.12	−0.28	89.30
Priapism	0.36	−0.20	−0.33	92.00
Burn with adherent objects to the skin	0.71	−0.09	−0.15	94.70
Facial burn	0.71	−0.15	−0.12	92.00
Non-compressible bleeding	0.23	−0.07	−0.33	96.00
Withdrawal signs	0.49	−0.04	−0.20	96.00
Severe dehydration signs	0.57	−0.15	−0.15	93.30
Acute testicular torsion	0.57	−0.15	−0.52	96.00
Labor	0.63	−0.17	−0.07	93.30
Closed abdominal trauma	0.47	−0.07	−0.33	89.30
Trauma with deformities	0.55	−0.04	−0.23	92.00
Trauma with penetrating objects in vital regions	0.65	−0.04	−0.33	84.00
Yellow Category				
Less than 15% of body surface area burned (second-degree)	0.60	−0.17	−0.23	96.00
Tonsillar abscess	0.55	−0.12	−0.20	96.00
Severe and acute test alterations	0.49	−0.09	−0.15	96.00
Avulsion of permanent tooth over two hours	0.55	−0.15	−0.23	93.30
Intermittent crying in a child under 2 years	0.57	−0.20	−0.23	89.30
Deformity of small bones	0.44	−0.12	−0.23	97.30
Jaw dislocation	0.52	−0.12	−0.31	94.70
Shoulder pain in pregnant women (Kehr’s sign)	0.47	−0.15	−0.25	92.00
Ventilation-dependent pain	0.49	−0.09	−0.17	94.70
Apparent mandibular edema	0.44	−0.07	−0.20	97.30
Periorbital edema with inflammatory signs	0.49	−0.09	−0.17	96.00
Significant genital edema	0.63	−0.12	−0.15	88.00
Muscular induration	0.33	−0.07	−0.28	88.00
Between 15% and 9% of body surface area burned (first-degree)	0.49	−0.17	−0.23	98.70
Reported frank hematuria	0.57	−0.12	−0.12	97.30
History of no urination in the last 24 hours	0.60	−0.09	−0.12	96.00
Suicidal ideation	0.60	−0.07	−0.20	98.70
Object insertion	0.47	−0.12	−0.17	96.00
Skin lesions suggestive of infectious diseases (blisters, petechiae)	0.47	−0.12	−0.12	89.30
Electrical injury	0.57	−0.09	−0.31	97.30
Suspected maltreatment injury	0.55	−0.15	−0.12	84.00
Movement limitation	0.39	−0.17	−0.07	93.30
Postictal state	0.41	−0.15	−0.25	98.70
Inflammatory process with serous, aqueous, or purulent exudate	0.63	−0.20	−0.09	96.00
Compressible bleeding	0.60	0.04	−0.17	96.00
Apparent dehydration signs	0.57	−0.12	−0.12	98.70
Inflammatory signs in the breast	0.63	−0.17	−0.04	94.70
Signs suggestive of sexual violence	0.49	−0.15	−0.12	93.30
Cough with hemoptysis	0.52	−0.07	−0.20	96.00
Eye trauma	0.55	−0.15	−0.23	92.00
Assault victim	0.47	−0.17	−0.12	93.30
Intermittent vomiting	0.49	−0.17	−0.12	96.00

*CVR – Content Validity Ratio.*

After the second round, the judges eliminated 42 of the 94 items, resulting in 52 items across the three risk categories. With 71 judges participating, the critical CVR value was set at 0.18. The results of the second round indicated that all items were considered valid regarding clarity, relevance, pertinence, and agreement with the severity classification (Table 3).

**Table 3:** Severity Assessment Items – Second Round of Evaluation of Items in the Urgency and Emergency Risk Classification Instrument (n = 71). São Paulo, Brazil, 2021

Instrument Items	Clarity (CVR)	Relevance (CVR)	Pertinence (CVR)	Concordance with Classification (%)
Red Category				
Traumatic amputation	0.97	0.97	0.97	98.60
Baby being born	0.73	0.88	0.94	98.60
Spinal column compromise	0.41	0.91	0.38	97.19
Convulsing	0.87	0.97	0.97	98.60
Unresponsive child	0.79	0.97	1.00	98.60
Open fracture of large parts	0.68	1.00	0.94	97.19
Witnessed gastrointestinal hemorrhage	0.55	0.94	0.83	97.19
Otorrhagia (Battle’s sign - Raccoon eyes)	0.49	0.94	0.88	97.19
Second- and third-degree burns in the perineal area	0.63	0.94	0.94	97.19
Local burns with exposure of systems or organs	0.65	1.00	0.97	98.60
Ineffective breathing	0.55	1.00	1.00	98.60
Active uncontrollable bleeding	0.97	0.97	1.00	97.19
Severe trauma	0.49	1.00	0.88	98.60
Non-patent airway	0.71	0.97	0.85	98.60
Orange Category				
Psychomotor agitation with risk of aggression	0.97	0.94	0.94	98.60
Neurological changes within 24 hours	0.55	1.00	0.80	98.60
Decreased fetal movements after one hour of active observation by the mother	0.60	0.88	0.57	98.60
Precordial pain	0.63	1.00	0.91	97.19
Postictal state	0.41	0.88	0.73	98.60
Vaginal bleeding in pregnant women over 20 weeks	0.60	0.94	0.82	98.60
Intense vaginal bleeding	0.39	0.88	0.80	95.78
Small part fracture	0.49	0.83	0.49	98.60
Inhalation of chemicals and/or smoke	0.68	0.97	0.69	98.60
Chemical and/or drug intoxication (licit and illicit)	0.88	0.94	0.74	98.60
Lacerating injury	0.47	1.00	0.94	98.60
Chemical injury	0.55	0.97	0.83	98.60
Severe ear pain with or without secretion	0.88	0.74	0.63	97.19
Associated precordial pain	0.41	0.94	0.83	97.19
Facial burn	0.71	0.97	1.00	97.19
Active non-compressible bleeding	0.74	0.85	0.68	97.19
Signs of alcohol and/or chemical withdrawal	0.91	0.71	0.74	97.19
Signs of meningism	0.74	0.91	0.67	98.60
Signs of closed abdominal trauma	0.88	0.97	0.36	95.78
Severe dehydration signs	0.57	1.00	0.80	97.19
Acute testicular torsion	0.57	0.85	0.21	98.60
Labor	0.97	0.88	0.88	97.19
Trauma with gross deformities	0.85	0.88	0.88	95.78
Trauma with penetrating objects in vital regions	0.97	0.97	1.00	94.37
Yellow Category				
Less than 15% of body surface area burned (second-degree)	0.60	0.85	0.91	98.60
Avulsion of permanent tooth over two hours	0.55	0.71	0.80	98.60
Between 15% and 9% of body surface area burned (first-degree)	0.49	0.80	0.91	98.60
Foreign body insertion	0.859	0.80	0.54	98.60
Suspected maltreatment injury	0.55	0.71	0.77	98.60
Acute movement limitation	0.39	0.88	0.82	98.60
Inflammatory process with serous, aqueous, or purulent exudate	0.63	0.60	0.91	97.19
Active compressible bleeding	0.746	0.79	0.91	95.78
Signs of tonsillar abscess	0.831	0.69	0.28	97.19
Inflammatory signs in the breast	0.857	0.68	0.83	98.60
Signs suggestive of sexual abuse	0.915	0.83	0.42	97.19
Signs suggestive of violence	0.859	0.80	0.85	97.19
Eye trauma	0.55	0.91	0.85	95.78
Intermittent vomiting	0.49	0.77	0.77	97.19

*CVR – Content Validity Ratio.*

## DISCUSSION

RC is an effective process for identifying potential severity situations, relating patient complaints and vital signs to ensure timely care^(1)^. The primary protocols used in our country are of international origin^(2, 7, 11)^. Therefore, providing a validated and applicable risk classification instrument for the profile of Brazilian public health service users of different age groups makes this study relevant, especially for PHC emergency services.

The risk classification instrument developed, after a literature review and retrospective analysis of the PHC attendance profile, was validated in the second cycle by a significant number of expert judges. It is intended as a facilitating tool for nurses in PHC emergency units and is tailored to the reality of PHC concerning material and human resources. In contrast, PHC is highlighted by the Urgency and Emergency Care Network as the element that organizes and coordinates the network^(5, 6)^; however, no risk classification instruments were found in the literature specifically directed to PHC emergency services.

Research on risk classifications most used in Brazil identified several studies on the MTS, which has five levels and follows a characteristic approach, with patient complaints and vital signs as the evaluation center^(2, 4, 12, 14, 18)^. However, there are significant difficulties related to the need for specific training for the Brazilian population and the challenge of direct contact with the Brazilian Group of Risk Classification, making long-term use financially unfeasible for many services, resulting in the discontinuation of the protocol. Nursing teams also emphasize the need for adaptations for specific care, such as mental health or vulnerability issues, as the MTS was designed for another country’s population^(11, 24, 25, 26, 27, 28)^.

In the context of risk classification proposed by the Ministry of Health, the HumanizaSUS stands out. This instrument comprises four risk levels, including guidance on how to perform classification and suggestions for some clinical situations related to severity. However, it does not propose waiting times for each level or a scale for vital signs or pain^(1)^. The absence of five levels creates difficulties for professionals when an intermediate care requirement arises. At this moment, he must choose whether to classify the patient as having an imminent emergency or a non-urgent priority, which may delay treatment and result in a potential adverse event^(14)^.

Thus, the developed risk classification instrument considers the profile of emergency care in PHC, focusing on symptoms, signs, and complaints related to the reason for seeking care, categorized into three severities: red, orange, and yellow. The goal is to reduce waiting times for individuals seeking care, tailored to the complexity level as well as the physical and human resources of the emergency service^(11, 24, 25, 26, 27, 28)^.

The second round’s results showed that all retained items had values above the cutoff point based on the number of judges. Thus, the instrument was validated in terms of clarity, relevance, pertinence, and agreement with the risk classification. The CVR obtained for relevance and pertinence was mainly above 0.80, with over 95% agreement regarding severity for all items.

Regarding the judges’ suggestions, all were accepted. The space for comments was essential for the process of improving the risk classification instrument. Concerning the item of burned body surface area, severe areas were identified, along with suggestions for improving the clarity of this item. Additionally, some items were removed following the feedback provided, making the instrument comprehensible, clear, and neither redundant nor cumbersome, thus making it applicable in this scenario.

A positive aspect was the participation of a significant number of judges (n = 75 and n = 71) from all Brazilian regions, unlike other studies with a similar approach but fewer participants^(29, 30)^. Another relevant point is the inclusion of items related to the population’s vulnerability, such as “Alcohol and/or chemical withdrawal symptoms,” “Trauma with penetrating objects in vital regions,” “Suspected maltreatment injuries,” “Signs suggestive of sexual abuse,” and “Signs suggestive of violence.” Such items had more than 97% agreement among judges concerning severity classification. These items were not found in the literature review, highlighting a unique aspect of the proposed instrument compared to protocols already used in our country.

### Study Limitations

The study’s limitations include the predominance of judges from the South and Southeast regions. Although content validity evidence was identified for all evaluated aspects, considering the different aspects assessed, there is a need for further evidence of the instrument’s internal structure validity.

### Contributions to the Field of Nursing

The content-validated risk classification instrument can be validated in the target population and subsequently serve as an appropriate tool for emergency services within the Primary Health Care context. This is because it was developed considering the complaints, main outcomes, and available resources in health establishments at this level of care. Using a risk classification instrument tailored to the patient profile enables nurses to have proper decision-making guidance and ensures safe and individualized decisions.

## CONCLUSION

The developed instrument demonstrated content validity evidence after two rounds of expert evaluation. It emerges as a current tool with potential use by nurses to safely perform risk classification in Primary Health Care emergency services.
