# Supplementary material for: Development and content validation of a risk classification instrument
Source: Rev Bras Enferm. 2024 Sep 6;77(4):e20230502. doi: 10.1590/0034-7167-2023-0502 (PMC11382668; doi:10.1590/0034-7167-2023-0502)
Supplement: Translation

# Translation: Desenvolvimento e validação de conteúdo de um Instrumento de classificação de risco

**DOI:** 10.1590/0034-7167-2023-0502pt

**Authors:** Marcia Beatriz Micha Ferreira de Oliveira, Lillian Caroline Fernandes, Ilana Eshriqui Oliveira, Ramon Antônio Oliveira, Flávio Rebustini, Ana Carolina Cintra Nunes Mafra, Eduarda Ribeiro dos Santos

**Affiliations:** I Sociedade Beneficente Israelita Brasileira Albert Einstein, Faculdade Israelita de Ciências da Saúde Albert Einstein. São Paulo, São Paulo, Brasil; II Universidade de São Paulo, Escola de Enfermagem, Departamento de Enfermagem Médico-Cirúrgica. São Paulo, São Paulo, Brasil; III Sociedade Beneficente Israelita Brasileira Albert Einstein, Centro de Estudos, Pesquisa e Práticas em Atenção Primária à Saúde e Redes (CEPPAR). São Paulo, São Paulo, Brasil; IV Universidade de São Paulo, Escola de Ciências, Humanidades e Artes, Departamento de Gerontologia. São Paulo, São Paulo, Brasil; V Hospital Sírio Libanês. São Paulo, São Paulo, Brasil

**Keywords:** Classificação de Risco, Estudo de Validação, Psicometria, Gravidade do Paciente, Atenção Primária a Saúde

## Abstract

**Objetivo:**

Desenvolver e validar o conteúdo de um instrumento para classificação de risco do paciente em serviços de emergência da Atenção Primária à Saúde.

**Método:**

Estudo composto por duas etapas: geração de itens e validade de conteúdo. Foi realizada revisão de literatura e análise retrospectiva dos prontuários para criação dos itens do instrumento. Na validação de conteúdo, foi usado o *Content Validity Ratio* (CVR) para averiguar a concordância entre os juízes.

**Resultados:**

O instrumento de classificação de risco foi validado por 75 e 71 juízes no primeiro e segundo ciclo, respectivamente. Foi utilizado escore mínimo da aderência ao item da variável latente com base no número final de juízes, adotando 0,22 e 0,18; e foram mantidos 52 itens divididos em três categorias de classificação (vermelho, laranja e amarelo).

**Conclusão:**

O instrumento foi considerado válido quanto à clareza, relevância, pertinência e concordância em relação à gravidade indicada no item.

## INTRODUÇÃO

Acolhimento é definido como prática de saúde por meio de escuta qualificada e da capacidade de pactuação entre a demanda do usuário e a possibilidade de resposta do serviço, devendo ser realizado por todos os profissionais. Em contraste, a Classificação de Risco (CR) é denominada como um processo de trabalho de caráter técnico e assistencial que prevê a celeridade no atendimento amparado por um protocolo pré-estabelecido. No Brasil, por meio da Resolução 423/2012, o Conselho Federal de Enfermagem determina que a CR é uma atividade privativa do profissional Enfermeiro^(1, 2, 3)^.

O alto volume de atendimento e consequente superlotação dos serviços de urgência e emergência são discutidos em cenário nacional e internacional^(4, 5)^. Consequentemente há constantes discussões acerca da Rede de Atenção à Saúde (RAS), especialmente sobre a Atenção Primária à Saúde (APS), a qual é indicada tanto como a porta de entrada quanto o elemento que ordena e coordena a Rede de Atenção de Urgência e Emergência. Nesse cenário, os protocolos de CR são reconhecidos como parte das resoluções assertivas relacionadas ao primeiro atendimento, fazendo-se imprescindível a sua implantação nos serviços de urgência e emergência da RAS^(5, 6)^.

Mundialmente, recomenda-se o emprego de protocolos que estratifiquem a gravidade clínica do paciente. Dentre eles, os mais usados são: *Australasian Triage Scale* (ATS), *Canadian Triage and Acuity Scale* (CTAS), *Emergency Severity Index* (ESI) e, de forma majoritária, o Sistema de Triagem Manchester (STM)^(7, 8, 9, 10, 11, 12, 13)^. O STM passou a ser o mais utilizado nos serviços de urgência e emergência em todos os níveis de atenção, porém, com o passar do tempo, algumas barreiras relacionadas ao custo começaram a ser percebidas no Brasil, pois sua implementação e manutenção demandam treinamento específico com o Grupo Brasileiro de Classificação de Risco^(13)^.

Alguns estudos demonstram que o STM foi um ótimo preditor de desfecho em serviços de emergência hospitalares^(14, 15)^. No entanto, em algumas pesquisas realizadas em instituições de atenção básica, foram evidenciadas fragilidades^(16, 17)^. Em um relato de experiência, é destacado que, devido ao STM ter sido desenvolvido em uma realidade hospitalar, alguns fatores como a baixa complexidade clínica e os recursos materiais das instituições de saúde da atenção básica dificultam a aplicabilidade do STM; por isso, podem ser necessárias algumas adaptações^(18)^. Esse achado foi endossado por uma revisão de escopo^(19)^.

Diante desse contexto, considerando que atualmente são utilizados instrumentos de classificação de risco desenvolvidos e validados especificamente para o cenário de cada país e voltados para a atenção hospitalar, torna-se necessário o desenvolvimento e a validação de um instrumento de classificação de risco para ser utilizado em serviços de emergência da Atenção Primária à Saúde do Sistema Único de Saúde^(13, 14, 15)^.

## OBJETIVO

Construir e validar um instrumento de classificação de risco para ser utilizado nos serviços de emergência da Atenção Primária à Saúde.

## MÉTODOS

### Aspectos éticos

O presente estudo foi aprovado pelo Comitê de Ética em Pesquisa com Seres Humanos da Secretaria Municipal da Saúde de São Paulo e pelo Comitê de Ética em Pesquisa com Seres Humanos da Sociedade Beneficente Israelita Brasileira Albert Einstein, via plataforma Brasil. Para fins de legitimação, todos os participantes assinaram o Termo de Consentimento Livre e Esclarecido (TCLE).

### Delineamento do estudo

Trata-se de um estudo psicométrico, constituído por duas etapas: geração de itens; e validade de conteúdo por meio de painel de especialistas. Foram seguidas as etapas de definição dos objetivos, construção dos itens, seleção e organização dos itens, estruturação do instrumento, opinião de especialistas e validade de conteúdo. Esta pesquisa foi estruturada de acordo com o *Revised Standards for Quality Improvement Reporting Excellence* (SQUIRE 2.0)^(20)^.

### Etapa 1: revisão bibliográfica e elaboração dos itens do instrumento

Para a construção do instrumento, realizou-se revisão narrativa nas bases de dados PubMed, LILACS, Scopus e Scielo utilizando os seguintes termos combinados com o operador booleano “OR”: Triagem, Classificação de Risco, HumanizaSUS e Manchester.

Além disso, nessa etapa, foi realizada análise exploratória retrospectiva das demandas dos serviços de emergência da APS. A APS do município de São Paulo está constituída por 450 Unidades Básicas de Saúde (UBS) e 117 unidades de Assistência Médica Ambulatorial (AMA), das quais 87 estão integradas a Unidades Básicas de Saúde^(21)^.

Em São Paulo, em 2005, as consultas de emergência representaram 43% de todas as consultas aos usuários do SUS, no entanto a maioria desses atendimentos eram de complexidade compatível com a UBS e não exigia a infraestrutura de pronto-socorro e hospitais. Diante desse cenário, em 2009, a Secretaria Municipal de Saúde propôs a criação das unidades de AMA, que estão vinculadas à atenção básica. Elas têm o objetivo de ampliar o acesso da população à rede básica de saúde^(22)^.

Considerando essas definições, foram consultadas as demandas de três unidades de AMA, as quais totalizaram 358.555 atendimentos em 2022. Foram analisados, de todos esses atendimentos, os sinais, sintomas e queixa principal relacionados a cada classificação de gravidade e aos desfechos de óbito e transferência. As variáveis categóricas foram descritas por frequência absoluta e porcentagens; e as variáveis numéricas, por média e desvio-padrão quando distribuição normal, ou por medianas e quartis, caso contrário. As relações entre cada uma das variáveis obtidas na CR com os desfechos foram analisadas por testes de hipóteses em relações dois a dois e por coeficientes de correlação, dependendo da natureza dos dados. As análises foram conduzidas no pacote computacional R Core Team com nível de significância de 5%.

Ao fim dessa etapa, foi verificado que a maioria dos atendimentos de emergência na APS são as categorias verde e azul, em que, pelos vigentes protocolos, os pacientes poderiam aguardar 120 e 240 minutos, respectivamente. A fim de garantir menor tempo de espera, o presente instrumento de classificação de risco dividiu os sinais e sintomas em três categorias: vermelho (atendimento imediato), laranja (atendimento em até 10 minutos) e amarelo (atendimento em até 60 minutos). Os instrumentos de classificação de risco são compostos por cinco categorias de gravidade: vermelho, laranja, amarelo, verde e azul, sugerindo tempo de espera de 0, 10, 50, 120 e 240 minutos, respectivamente.

### Etapa 2: validação de conteúdo

Foram elencados os seguintes critérios de inclusão: i) ser médico ou enfermeiro com pelo menos cinco anos de formação, e ii) atuar em serviços públicos brasileiros de urgência e emergência; unidade de tratamento intensiva; APS; ou docência universitária com experiência em pesquisa na área de urgência e emergência. A identificação e seleção de especialistas iniciou com a busca no sistema de Curriculum Lattes, utilizando filtros de área de atuação profissional e selecionando: grande área – ciências da saúde; área – enfermagem e medicina; e subárea – “urgência e emergência” e “emergência”.

O convite das duas rodadas foi realizado por meio da técnica “bola de neve”^(23)^, de modo que o contato foi feito com profissionais elegíveis de todos os estados brasileiros, os quais poderiam indicar outros possíveis juízes. O link com o convite para participação da pesquisa como juiz foi encaminhado via grupos, divulgação em redes sociais, nos sites do Conselho Federal de Enfermagem e Conselho Regional de Enfermagem – São Paulo. O processo de validação aconteceu em dois períodos: primeira rodada de janeiro a março de 2021 e segunda rodada de julho a setembro de 2021.

### Coleta de dados

Após verificar os critérios de elegibilidade, foi enviado o TCLE via e-mail e o link de acesso ao formulário para avaliação do instrumento de CR, acompanhado de um arquivo com informações detalhadas e/ou imagens sobre os itens propostos para validação (material suplementar). A etapa de coleta de dados foi realizada por meio do RedCap®.

Os itens foram avaliados pelos juízes em relação aos domínios de: i) clareza, ii) relevância, iii) pertinência e iv) concordância quanto à classificação de gravidade do item. Havia ainda a possibilidade de realizar sugestões de redação para cada item.

Antes do questionário, foram disponibilizadas afirmativas a serem analisadas para cada item: i) “O item está claro: considerando a forma como está escrito, é possível identificar o que precisa ser avaliado”; ii) “O item é relevante para medir a gravidade do paciente: é fundamental que o item seja considerado na avaliação da classificação de risco”; iii) “O item é mensurável pelo enfermeiro: na prática, é possível que o enfermeiro consiga fazer a avaliação desse item no momento da classificação de risco; iv) “Este item precisa mudar de classificação: o item é relevante e pertinente, porém está na classificação errada. Caso queira deixar algum comentário ou sugerir alguma mudança, basta clicar em ‘comentar’, que será aberto um campo de texto livre para cada item ao final de cada classificação”. Para cada domínio, foi utilizado um layout com as opções de “sim” ou “não” para avaliação de todos os itens. O questionário foi considerado válido quando houve resposta em todos os itens e domínios.

### Análise de dados

Optou-se pelo método de cálculo de *Content Validity Ratio* (CVR), que propõe validar os itens de um questionário ou instrumento com base na concordância de um painel amplo de especialistas sobre os critérios avaliados de cada item, variando de -1 (discordância perfeita) a 1 (concordância perfeita)^(18, 19)^. Os coeficientes de concordância foram comparados à classificação presente em *Altman,* que considera como ruins os coeficientes menores que 0,2; razoáveis aqueles entre 0,2 e 0,4; moderados aqueles entre 0,4 e 0,6; bons entre 0,6 e 0,8; e excelentes aqueles acima de 0,8. O escore mínimo da aderência do item à variável latente foi baseado nos valores críticos do CVR tendo por base o número final de juízes, adotando 0,22 como ponto de corte na primeira rodada e 0,18 na segunda para considerar o item satisfatório do ponto de vista da validade do seu conteúdo^(18, 19)^. As análises estatísticas foram realizadas no software Statistical Package for the Social Sciences (SPSS).

## RESULTADOS

### Etapa 1: caracterização dos juízes

O instrumento proposto foi avaliado em duas rodadas até obter o nível necessário de consenso. O perfil dos juízes participantes encontra-se descrito na Tabela 1.

**Tabela 1:** Caracterização dos juízes especialistas participantes nas duas rodadas. São Paulo, Brasil, 2021

Dados de caracterização	Primeira rodada (n = 75)	Segunda rodada (n = 71)
Média da idade em anos (DP) Variáveis categóricas: nº (%)	39,95 (8,00)	42 (8,00)
Sexo		
Feminino	54 (72,00)	48 (67,60)
Masculino	21 (28,00)	23 (32,40)
Profissão		
Médico	15 (20,00)	11 (15,50)
Enfermeiro	60 (80,00)	60 (84,50)
Último grau de formação		
Graduação	8 (10,67)	9 (12,70)
Residência	4 (5,33)	2 (2,80)
Especialização	49 (65,33)	43 (60,60)
Mestrado	6 (8,00)	12 (16,90)
Doutorado	6 (8,00)	5 (7,00)
Pós-doutorado	2 (2,67%)	0 (0,00)
Região brasileira de atuação profissional		
Norte	1 (1,33)	1 (1,40)
Nordeste	1 (1,33)	2 (2,80)
Centro-Oeste	6 (8,00)	2 (2,80)
Sul	11(14,67)	10 (14,10)
Sudeste	56 (74,67)	56 (78,90)
Área de atuação ou pesquisa*		
Urgência e Emergência	38 (50,67)	42 (59,20)
Atenção Primária	40 (53,33)	29 (40,80)
Assistência de Enfermagem em cenário hospitalar	16 (21,33)	24 (33,80)
Obstetrícia	1 (1,33)	2 (2,8)
Pediatria	12 (16,00)	5 (7,0)

*Foi possível assinalar mais de uma opção; DP – desvio padrão.

### Etapa 2: construção dos itens

Após a revisão bibliográfica e análise dos dados na etapa exploratória retrospectiva, considerando as queixas com maior frequência e as queixas relacionadas a desfechos de transferência, óbito e alta, o instrumento foi composto por 94 itens, sendo distribuídos nas três categorias de gravidade (vermelho, laranja e amarelo). Na primeira rodada, todos os itens apresentaram CVR superior ao valor crítico para o domínio “Clareza”, porém, nos demais itens e domínios, não foi atingido o ponto de corte (Tabela 2).

**Tabela 2:** Primeira rodada de avaliação dos itens do instrumento de classificação de risco em urgência e emergência (n=75). São Paulo, Brasil, 2021

Itens do instrumento de classificação de risco	Clareza (CVR)	Relevância (CVR)	Pertinência (CVR)	Concordância com a classificação (%)
Categoria: Vermelho				
Acima de 25% da superfície corpórea queimada	0,41	−0,07	−0,17	98,70
Amputação traumática	0,68	−0,17	−0,23	98,70
Bebê nascendo	0,73	−0,33	−0,23	98,70
Comprometimento de coluna vertebral	0,41	−0,12	−0,52	97,30
Convulsionando	0,87	−0,23	−0,25	100,00
Criança não responsiva	0,79	−0,17	−0,33	98,70
Fratura exposta de grandes partes	0,68	−0,15	−0,28	100,00
Hemorragia digestiva presenciada	0,55	−0,17	−0,28	100,00
Otorragia (Sinais de Battle - Guaxinim)	0,49	−0,12	−0,31	100,00
Queimadura em face acometendo vias aéreas	0,73	−0,09	−0,23	100,00
Queimaduras de 2° e 3° grau em região de períneo	0,63	−0,20	−0,23	100,00
Queimaduras locais com exposição de sistema ou órgãos	0,65	−0,17	−0,28	100,00
Respiração ineficaz	0,55	−0,17	−0,28	96,00
Sangramento incontrolável	0,65	−0,15	−0,28	98,70
Trauma grave	0,49	−0,12	−0,25	100,00
Via aérea não pérvia	0,71	−0,15	−0,31	100,00
Categoria: Laranja				
Abaulamento de fontanela	0,68	−0,07	−0,20	96,00
Agitação psicomotora	0,60	−0,09	−0,12	98,70
Alteração visual súbita	0,57	−0,07	−0,33	96,00
Alterações neurológicas em menos de 24 horas	0,55	−0,15	−0,28	96,00
Amputação de pequenas partes	0,60	−0,04	−0,12	94,70
Avulsão de dente permanente em menos de duas horas	0,36	−0,23	−0,31	93,30
Bexigoma	0,55	−0,20	−0,12	93,30
Criança prostrada	0,52	−0,12	−0,15	97,30
Desconforto respiratório evidente	0,60	−0,01	−0,20	92,00
Diminuição de movimentos fetais	0,60	−0,17	−0,12	94,70
Dor em precórdio	0,63	−0,04	−0,31	89,30
Enterorragia	0,63	−0,09	−0,25	98,70
Entre 24% e 15% de superfície corpórea queimada	0,55	−0,12	−0,20	96,00
Epistaxe presenciada	0,65	−0,12	−0,07	98,70
Estado pós-ictal	0,41	−0,07	−0,28	96,00
Fluxo sanguíneo vaginal em gestante com mais de 20 semanas	0,60	−0,20	−0,20	96,00
Fluxo sanguíneo vaginal intenso	0,39	−0,25	−0,12	88,00
Fratura de pequenas partes	0,49	−0,09	−0,36	97,30
Hálito cetônico	0,71	−0,09	−0,20	97,30
Inalação de agentes químicos	0,63	−0,04	−0,36	97,30
Inalação de fumaça	0,68	0,01	−0,33	96,00
Inserção de objeto potencialmente perigoso	0,52	−0,17	−0,23	93,30
Instabilidade da pelve	0,49	−0,04	−0,39	89,30
Intoxicação alcoólica	0,55	−0,09	−0,23	97,30
Intoxicação por drogas	0,57	−0,09	−0,28	97,30
Intoxicação por produtos químicos	0,60	−0,12	−0,31	96,00
Lesão dilacerante	0,47	−0,01	−0,25	93,30
Lesão por agente químico	0,55	−0,01	−0,25	96,00
Lesão por veneno letal conhecido	0,60	−0,01	−0,41	88,00
Lesão vascular arterial ou venosa	0,55	−0,12	−0,39	94,70
Melena	0,60	−0,15	−0,33	98,70
Meningismo	0,60	−0,04	−0,15	96,00
Otalgia intensa	0,63	−0,01	−0,20	96,00
Perda de líquido amniótico	0,55	−0,25	−0,20	94,70
Precordialgia *associada*	0,41	−0,12	−0,28	89,30
Priapismo	0,36	−0,20	−0,33	92,00
Queimadura com presença de objetos aderidos à pele	0,71	−0,09	−0,15	94,70
Queimadura em face	0,71	−0,15	−0,12	92,00
Sangramento não compressível	0,23	−0,07	−0,33	96,00
Sinais de abstinência	0,49	−0,04	−0,20	96,00
Sinais graves de desidratação	0,57	−0,15	−0,15	93,30
Torção testicular aguda	0,57	−0,15	−0,52	96,00
Trabalho de parto	0,63	−0,17	−0,07	93,30
Trauma abdominal fechado	0,47	−0,07	−0,33	89,30
Trauma com deformidades	0,55	−0,04	−0,23	92,00
Trauma com objetos penetrantes em regiões vitais	0,65	−0,04	−0,33	84,00
Categoria: Amarelo				
Abaixo de 15% da superfície corpórea queimada de 2° grau	0,60	−0,17	−0,23	96,00
Abcesso amigdaliano	0,55	−0,12	−0,20	96,00
Alterações de exames graves e agudas	0,49	−0,09	−0,15	96,00
Avulsão de dente permanente há mais de duas horas	0,55	−0,15	−0,23	93,30
Choro intermitente em criança de até 2 anos	0,57	−0,20	−0,23	89,30
Deformidade de ossos menores	0,44	−0,12	−0,23	97,30
Deslocamento de mandíbula	0,52	−0,12	−0,31	94,70
Dor irradiada para o ombro em gestante (sinal de Kehr)	0,47	−0,15	−0,25	92,00
Dor ventilatório-dependente	0,49	−0,09	−0,17	94,70
Edema aparente em região mandibular	0,44	−0,07	−0,20	97,30
Edema em região periorbitária com sinais flogísticos	0,49	−0,09	−0,17	96,00
Edema importante em órgão genital	0,63	−0,12	−0,15	88,00
Empastamento muscular	0,33	−0,07	−0,28	88,00
Entre 15% e 9% da superfície corpórea queimada de 1° grau	0,49	−0,17	−0,23	98,70
Hematúria franca referida	0,57	−0,12	−0,12	97,30
História de ausência de urina nas últimas 24 horas	0,60	−0,09	−0,12	96,00
Ideação suicida	0,60	−0,07	−0,20	98,70
Inserção de objeto	0,47	−0,12	−0,17	96,00
Lesão de pele com características sugestivas de doenças infectocontagiosas (manchas em bolhas cheias de líquido e/ou petéquias)	0,47	−0,12	−0,12	89,30
Lesão elétrica	0,57	−0,09	−0,31	97,30
Lesão sugestiva de maus-tratos	0,55	−0,15	−0,12	84,00
Limitação do movimento	0,39	−0,17	−0,07	93,30
Pós-comicial	0,41	−0,15	−0,25	98,70
Processo inflamatório com presença de exsudato seroso, aquoso ou purulento	0,63	−0,20	−0,09	96,00
Sangramento compressível	0,60	0,04	−0,17	96,00
Sinais aparentes de desidratação	0,57	−0,12	−0,12	98,70
Sinais flogísticos em mama	0,63	−0,17	−0,04	94,70
Sinais sugestivos de violência sexual	0,49	−0,15	−0,12	93,30
Tosse com hemoptise	0,52	−0,07	−0,20	96,00
Trauma ocular	0,55	−0,15	−0,23	92,00
Vítima de agressão	0,47	−0,17	−0,12	93,30
Vômitos intermitentes	0,49	−0,17	−0,12	96,00

*CVR – Content Validity Ratio.*

Após a segunda rodada, 42 dos 94 itens foram eliminados, resultando em 52 itens, distribuídos nas três categorias de risco. Com a participação de 71 juízes, foi estabelecido o valor de 0,18 como crítico do CVR. Os resultados da segunda rodada indicaram que todos os itens foram considerados válidos quanto à clareza, relevância, pertinência e concordância no tocante à classificação de gravidade do item (Tabela 3).

**Tabela 3:** Itens de avaliação de gravidade – Segunda rodada de avaliação dos itens do instrumento de classificação de risco em urgência e emergência (n = 71). São Paulo, Brasil, 2021

Itens do instrumento	Clareza (CVR)	Relevância (CVR)	Pertinência (CVR)	Concordância com a classificação (%)
Categoria: Vermelho				
Amputação causada por trauma abrupto	0,97	0,97	0,97	98,60
Bebê nascendo	0,73	0,88	0,94	98,60
Comprometimento de coluna vertebral	0,41	0,91	0,38	97,19
Convulsionando	0,87	0,97	0,97	98,60
Criança não responsiva	0,79	0,97	1,00	98,60
Fratura exposta de grandes partes	0,68	1,00	0,94	97,19
Hemorragia digestiva presenciada	0,55	0,94	0,83	97,19
Otorragia (Sinais de Battle – Guaxinim)	0,49	0,94	0,88	97,19
Queimaduras de 2º e 3º grau em região de períneo	0,63	0,94	0,94	97,19
Queimaduras locais com exposição de sistemas ou órgãos	0,65	1,00	0,97	98,60
Respiração ineficaz	0,55	1,00	1,00	98,60
Sangramento incontrolável ativo	0,97	0,97	1,00	97,19
Trauma grave	0,49	1,00	0,88	98,60
Via aérea não pérvia	0,71	0,97	0,85	98,60
Categoria: Laranja				
Agitação psicomotora com risco de agressão	0,97	0,94	0,94	98,60
Alterações neurológicas em menos de 24 horas	0,55	1,00	0,80	98,60
Diminuição de movimentos fetais após uma hora de observação ativa pela mãe	0,60	0,88	0,57	98,60
Dor em precórdio	0,63	1,00	0,91	97,19
Estado pós-ictal	0,41	0,88	0,73	98,60
Fluxo sanguíneo vaginal em gestantes com mais de 20 semanas	0,60	0,94	0,82	98,60
Fluxo sanguíneo vaginal intenso	0,39	0,88	0,80	95,78
Fratura de pequenas partes	0,49	0,83	0,49	98,60
Inalação de agentes químicos e/ou fumaça	0,68	0,97	0,69	98,60
Intoxicação por produtos químicos e/ou drogas lícitas e ilícitas	0,88	0,94	0,74	98,60
Lesão dilacerante	0,47	1,00	0,94	98,60
Lesão por agente químico	0,55	0,97	0,83	98,60
Otalgia intensa com ou sem presença de secreção	0,88	0,74	0,63	97,19
Precordialgia associada	0,41	0,94	0,83	97,19
Queimadura em face	0,71	0,97	1,00	97,19
Sangramento não compreensível ativo	0,74	0,85	0,68	97,19
Sinais de abstinência alcoólica e/ou química	0,91	0,71	0,74	97,19
Sinais de meningismo	0,74	0,91	0,67	98,60
Sinais de trauma abdominal fechado	0,88	0,97	0,36	95,78
Sinais graves de desidratação	0,57	1,00	0,80	97,19
Torção testicular aguda	0,57	0,85	0,21	98,60
Trabalho de parto	0,97	0,88	0,88	97,19
Trauma com deformidades grosseiras	0,85	0,88	0,88	95,78
Trauma com objetos penetrantes em regiões vitais	0,97	0,97	1,00	94,37
Categoria Amarelo				
Abaixo de 15% da superfície corpórea queimada de 2° grau	0,60	0,85	0,91	98,60
Avulsão de dente permanente há mais de duas horas	0,55	0,71	0,80	98,60
Entre 15% e 9% da superfície corpórea queimada de 1° grau	0,49	0,80	0,91	98,60
Inserção de corpo estranho	0,859	0,80	0,54	98,60
Lesão sugestiva de maus-tratos	0,55	0,71	0,77	98,60
Limitação aguda de movimento	0,39	0,88	0,82	98,60
Processo inflamatório com presença de exsudato seroso, aquoso ou purulento	0,63	0,60	0,91	97,19
Sangramento compressível ativo	0,746	0,79	0,91	95,78
Sinais de abcesso amigdaliano	0,831	0,69	0,28	97,19
Sinais flogísticos em mama	0,857	0,68	0,83	98,60
Sinais sugestivos de abuso sexual	0,915	0,83	0,42	97,19
Sinais sugestivos de vítima de violência	0,859	0,80	0,85	97,19
Trauma ocular	0,55	0,91	0,85	95,78
Vômitos intermitentes	0,49	0,77	0,77	97,19

*CVR – Content Validity Ratio*

## DISCUSSÃO

Entende-se a CR como processo eficaz para identificação de possíveis situações de gravidade relacionando a queixa e sinais vitais do paciente, assim proporcionando atendimento em tempo adequado^(1)^. Os principais protocolos utilizados em nosso país são de origem internacional^(2, 7, 11)^. Logo, disponibilizar um instrumento de classificação de risco, com conteúdo validado e aplicável ao perfil dos usuários de serviços de saúde públicos brasileiros de diferentes faixas etárias torna este estudo relevante, especialmente aos serviços de emergência da APS. O instrumento de classificação de risco desenvolvido, após busca na literatura e análise retrospectiva do perfil de atendimentos da APS, foi validado no segundo ciclo por um número significativo de juízes especialistas na área.

O instrumento de classificação de risco é proposto como uma ferramenta facilitadora para enfermeiros que atendem nas unidades de serviços de emergência da APS de forma universal; e é voltado para a realidade da APS no tocante a recursos materiais e humanos. De forma contrastante, a APS é apontada pela Rede de Urgência e Emergência como o elemento que ordena e coordena a rede^(5, 6)^; entretanto, não foram encontrados na literatura instrumentos de classificação de risco direcionados aos serviços de emergência da APS.

Ao investigar as classificações de risco mais utilizadas no Brasil, foram encontrados diversos estudos sobre o Sistema de Triagem de Manchester, protocolo este que conta com cinco níveis e segue uma abordagem característica, sendo a queixa do paciente e os sinais vitais o centro da avaliação^(2, 4, 12, 14, 18)^. No entanto, observam-se grandes dificuldades relacionadas à necessidade de ter um treinamento específico para população brasileira e à dificuldade de contato direto com o Grupo Brasileiro de Classificação de Risco, o que para muitos serviços torna-se financeiramente inviável em longo prazo e resulta na descontinuidade da utilização do protocolo. As equipes de enfermagem também ressaltam necessidades de adaptações para atendimentos específicos, como questões de saúde mental ou vulnerabilidade, visto que o Sistema de Triagem Manchester foi idealizado para uma população de outro país^(11, 24, 25, 26, 27, 28)^.

No contexto da classificação de risco proposta pelo Ministério da Saúde, destaca-se o HumanizaSUS. Esse instrumento é composto por quatro níveis de risco, contando com orientações de como realizar a classificação e sugestões de algumas situações clínicas que podem ser relacionadas com a gravidade. Contudo, não propõe o tempo de atendimento para cada nível nem escala de sinais vitais ou dor^(1)^. Observa-se que não contar com cinco níveis gera dificuldade ao profissional quando existe um atendimento que requer cuidados intermediários. Nesse momento, ele precisa escolher incluir o paciente em uma classificação de emergência iminente ou prioridade não urgente, o que pode acarretar o atraso do tratamento e um desfecho com potencial evento adverso^(14)^.

Dessa forma, o instrumento de classificação de risco foi desenvolvido considerando o perfil de atendimentos de emergência da APS, focalizando sinais, sintomas e queixas relacionadas ao motivo da busca pelo atendimento, com categorização em três gravidades: vermelho, laranja e amarelo. O objetivo é diminuir o tempo de espera do indivíduo que busca atendimento, voltan-do-se para o nível de complexidade, bem como recursos físicos e humanos do serviço de emergência^(11, 24, 25, 26, 27, 28)^.

Evidenciou-se que, na segunda rodada, todos os itens mantidos tiveram valor superior ao ponto de corte, baseado no número de juízes. Dessa forma, o instrumento foi considerado validado em relação à clareza, relevância, pertinência e concordância com a classificação de risco. Além disso, destaca-se que o CVR obtido quanto à relevância e pertinência foi majoritariamente superior a 0,80 e todos os itens tiveram valor superior a 95% em relação à concordância com a gravidade obtida.

No tocante às sugestões dos juízes, todas foram acatadas. O espaço para comentários foi essencial para o processo de melhoria no instrumento de classificação de risco. Em relação ao item de superfície corpórea queimada, foram apontadas áreas consideradas graves, além de sugestões de melhorias em relação à clareza desse item. Ademais, alguns itens foram retirados após os apontamentos realizados, tornando o instrumento compreensível, claro e não redundante nem cansativo, tornando-o aplicável neste cenário.

Um ponto a ser ressaltado positivamente refere-se ao painel de juízes com uma quantidade expressiva (n = 75 e n = 71), oriundos das cinco regiões geográficas do Brasil, o que difere de estudos com abordagem semelhante que tiveram menor quantitativo nas respectivas amostras^(29, 30)^. Outro ponto relevante trata dos itens relacionados a situações de vulnerabilidade da população-alvo deste instrumento, como “Sinais de abstinência alcóolica e/ou química”, “Trauma com objetos penetrantes em regiões vitais”, “Lesão sugestiva de maus-tratos”, “Sinais sugestivos de abuso sexual” e “Sinais sugestivos de vítima de violência”. Tais itens apresentaram mais de 97% de concordância entre os juízes quanto à classificação de gravidade. Vale dizer que esses itens não foram encontrados durante o processo de revisão de literatura, destacando-se, assim, um diferencial do instrumento proposto em relação aos protocolos já utilizados em nosso país.

### Limitações do estudo

Como limitação, aponta-se a predominância de juízes das regiões Sul e Sudeste. Foi identificada a evidência de validade de conteúdo para todos os itens, considerando os diferentes aspectos avaliados, porém salienta-se a necessidade de obter evidências de validade de estrutura interna do instrumento.

### Contribuições para a área de Enfermagem

O instrumento de classificação de risco com evidências de validade de conteúdo pode ser validado na população-alvo e, posteriormente, representar uma ferramenta adequada para os serviços de emergência inseridos no contexto da atenção básica de saúde. Isso porque ele foi desenvolvido considerando as queixas, os principais desfechos e os recursos disponíveis em estabelecimentos de saúde desse nível de atenção. A utilização de um instrumento de classificação de risco de acordo com o perfil de paciente possibilita que o profissional enfermeiro tenha um direcionamento adequado da conduta decisória e uma tomada de decisão segura e individualizada.

## CONCLUSÃO

O instrumento desenvolvido mostrou evidências de validade de conteúdo após duas rodadas de apreciação pelos juízes. Ele desponta como uma ferramenta atual com potencial de uso pelos enfermeiros para realizar com segurança a classificação de risco nos serviços de emergência da Atenção Primária à Saúde.
